# Endovascular repair of aneurysms of the abdominal aorta: patients with hostile anatomy of the aneurysm neck

**DOI:** 10.3389/fsurg.2025.1648925

**Published:** 2025-12-10

**Authors:** E. L. Kalmykov, I. Droc, G. Kheladze, I. A. Suchkov, R. Dammrau

**Affiliations:** 1 Department of Vascular Surgery, Brandenburg Medical School, University Hospital Brandenburg/Havel, Brandenburg, Germany; 2 Central Military Hospital, Cardiovascular Surgery, Bucharest, Romania; 3 Department and Medical Centre for High Technology, University Hospital, Tbilisi State Medical University, Tbilisi, Georgia; 4 Department of Cardiovascular, Endovascular Surgery and Diagnostic Radiology, Ryazan State Medical University, Ryazan, Russia; 5 Department of Endovascular Aortic Therapy, Helios Clinic, Siegburg, Germany

**Keywords:** abdominal aortic aneurysm, hostile neck, endoleak, aneurysm neck angulation, aneurysm neck length, mortality

## Abstract

**Introduction:**

In some cases, endovascular abdominal aortic repair (EVAR) may be hampered by specific anatomical features, the so-called “hostile neck.”

**Aim:**

To analyse the results after implanting Gore excluder conformable endograft prostheses in patients with hostile neck anatomy who had experienced an infrarenal aneurysm.

**Methods:**

This is a retrospective, multicentre study that included 30 patients who were treated between April 2019 and August 2023. Primary endpoints were technical success, immediate results with intraoperative mortality and rate of endoleaks, short-term results with 30-day mortality, complications, endoleaks, migration rate, and mid-term follow-up.

**Results:**

EVAR was performed in 29 (96%) patients as an elective surgery and in one patient as emergency treatment. The median age of the patients was 75.0 years (range 71.0–79.0); of them, 83.3% were men. The median diameter of the aneurysm was 58.5 mm (range 55.0–66.0 mm). The median angle of the neck of the aortic aneurysm was 80.0° (range 30.0–90.0), with a median length of 15.0 mm (range 10.0–21.0). Of the 30 patients, seven fell outside the standard instructions for use. The technical success rate of the procedure was 100%. In three patients, one of the renal arteries was stented using the chimney technique. No type Ia or Ib endoleak was detected intraoperatively. In one patient, acute thrombosis of the graft was diagnosed after 21 days, requiring graft explantation. The 30-day mortality rate was 3.3%. No endoleaks of types Ia or Ib were identified before discharge from hospital. An endoleak of type II was identified in four patients. No local complications were identified. The mean follow-up period was 25.5 months (range 6.0–36 months). No further intervention was required. No late aortic rupture, endoprosthesis migration, or death was subsequently observed.

**Conclusion:**

The use of Gore excluder conformable endoprostheses is an effective method to treat abdominal aortic aneurysms with hostile neck, with good mid-term results; however, further research on long-term results is necessary.

## Introduction

The last three decades have been marked by the widespread use of endovascular abdominal aortic repair (EVAR) in the treatment of patients with infrarenal abdominal aortic aneurysm (iAAA), significantly improving patient outcomes. However, the procedure can be limited by challenging anatomical features, commonly referred to as “hostile neck” anatomy, such as a neck length <10 mm, neck angulation >60°, ≥50% circumferential thrombus at least 2 mm thick in the proximal neck (1). Clinical experience with many grafts in patients with hostile necks remains limited, and in practice, grafts are often used outside their instructions for use (IFU) (2), or more complex surgical techniques are employed. Using endografts beyond IFU recommendations has been associated with higher rates of early and late type I endoleak (1–9), early interventions, and late death. In some cases, progression of type I endoleak after endoprosthetics can result in aneurysm rupture, necessitating open conversion—a procedure associated with high morbidity and mortality. However, vascular surgeons now have access to a new endograft that may be implanted in patients with complex anatomy of the neck of an abdominal aortic aneurysm (10–16). In this context, our objective was to demonstrate the medium-term results of implanting a Gore excluder conformable endograft with Active Control System (GECE, W. L. Gore & Associates, Flagstaff, AZ, USA) in patients with hostile neck anatomy of an infrarenal aneurysm. The conformable excluder is an enhanced version of the Gore excluder, which has long been available on the market. The conformability allows its use in more challenging neck anatomies.

## Methods

### Study design

This is a retrospective, multicentre study designed to evaluate the experience und outcomes of using the GECE with Active Control System for the treatment of iAAA in patients with short and highly angulated infrarenal aortic necks. The GECE expands the applicability of EVAR up to 90° and lengths down to 10 mm. This innovative system allows the surgeon to angulate and reposition the device intraoperatively to optimise conformability and maximise sealing during implantation.

Our study included 30 patients. The patients were treated between April 2019 and August 2023 in the following centres: Department of Cardiovascular Surgery, Central Military Hospital, Bucharest, Romania; Department and Medical Centre for High Technology, University Hospital, Tbilisi State Medical University, Tbilisi, Georgia; Department of Endovascular Aortic Therapy, Helios Clinic, Siegburg, Germany. Our study was performed in line with the requirements of the local ethics committee of all clinics and adhered to the Declaration of Helsinki.

Primary endpoints were in accordance with the reporting standards of the Society for Vascular Surgery (17).

All patients were classified as being inside or outside the IFU, as based on the IFU of the GECE device.

#### Statistical analysis

The statistical analysis was conducted using the Statistica 12 application program (StatSoft Inc., Tulsa, OK, USA). Absolute values are presented as mean ± SD (or medians with quartiles [25q–75q]) and relative values are presented as percentages (%).

## Results

Demographic data are presented in Table 1. Graft implantation was performed in 29 (96%) patients as an elective operation and in one patient as an emergency procedure for a ruptured iAAA.

**Table 1:** Demographic data.

Overall	*n*	%
Age, years, median 75.0 [71.0; 79.0]		
Sex, *n* (%)		
Male	25	83.3
Female	5	16.7
Hypertension, *n* (%)	25	83.3
Smoking, *n* (%)	14	46.7
Coronary artery disease, *n* (%)	8	26.7
RD, *n* (%)	3	10.0
Diabetes *n* (%)	2	6.7
COPD, *n* (%)	1	3.3
Peripheral arterial disease, *n* (%)	2	6.7
Aneurysm diameter in mm	58.5 [55.0; 66.0]	
Aortic aneurysm neck angle (degrees)	80.0 [30.0; 90.0]	
Length of neck aneurysm in mm	15.0 [10.0; 21.0]	

Overall numbers (*n*) and percentages median, and 25–75 quartiles are given for patient age and geometry of the aneurysm.

### Vascular access

EVAR was performed using the percutaneous Manta closure device (Teleflex Incorporated, Wayne, PA, USA) in 21 (70%) patients, and using the cutdown technique in the remaining patients.

Of the 30 patients, seven were treated outside the standard IFU: four due to insufficient neck length and three due to excessive neck angulation.

**Figure 1:**
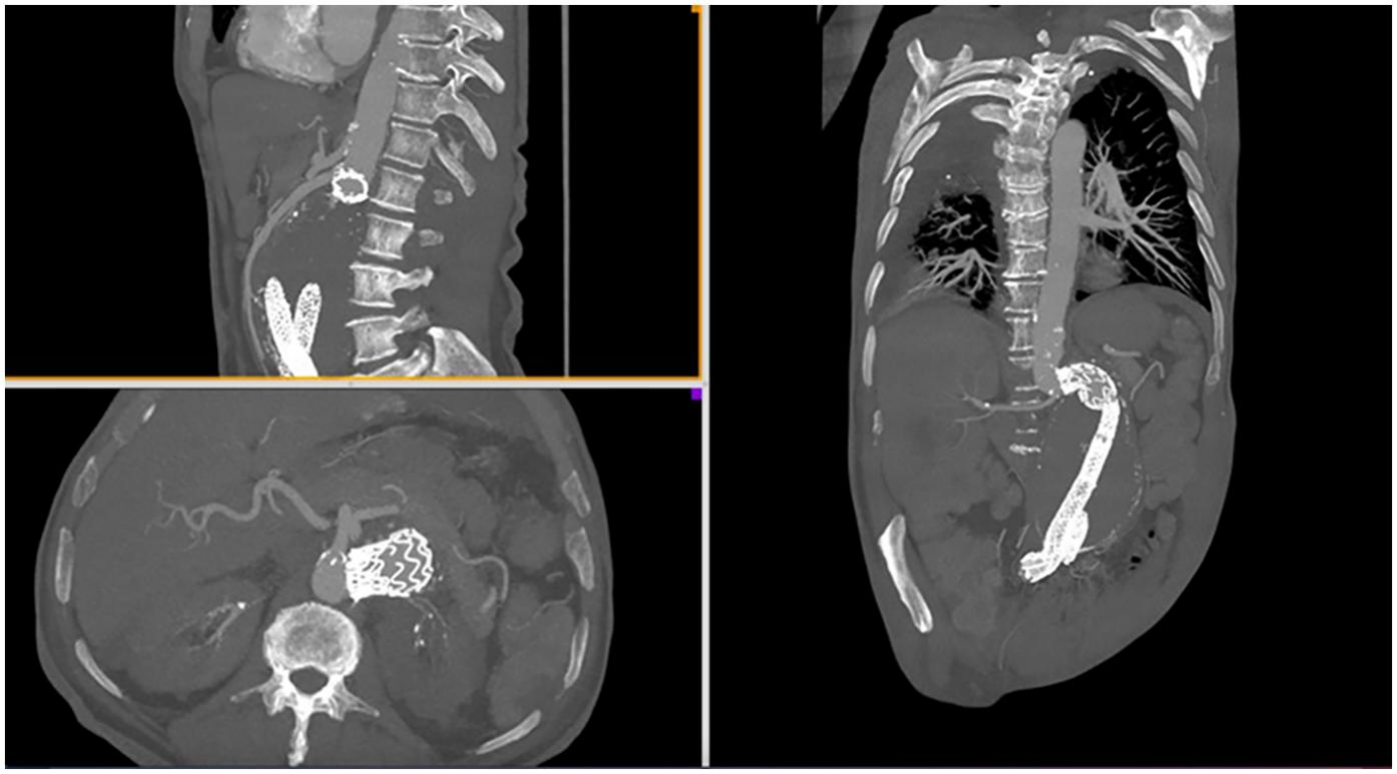
CT angiography imaging of a patient after graft implantation with aortic neck angulation of 110° and neck length of 10 mm.

Implantation of the GECE was performed in patients with a median aortic neck angulation of 80.0° (range 30.0–90.0), and a median length of 15.0 mm (range 10.0–21.0). Figure 1 illustrates a case in which the graft was implanted in a patient with an aortic neck angulation of 110° and a neck length of 10 mm, achieving good results. The postoperative CT scan shows no type I endoleak; the prosthesis is entirely adjacent to the aneurysm neck wall, with no evidence of endoleak or migration after 12 months of follow-up.

### Technical success

The technical success rate of the procedure was 100%. In three patients, one of the renal arteries was stented using the chimney technique, and in one patient, an additional stent was placed in the renal artery due to critical stenosis. In 12 patients, two rotational manoeuvres were performed, with repositioning required in three patients.

### Immediate results

No type Ia or Ib endoleak was detected intraoperatively. In one patient, acute graft thrombosis, likely infection-related, was diagnosed 21 days postoperatively and presented with ischemia, necessitating explantation and replacement with a Dacron bifurcated prosthesis. No complications were observed after the use of the Manta closure device.

### Short-term results

The 30-day mortality rate was 3.3%. One patient died from multiorgan failure after aneurysm rupture. No type Ia or Ib endoleaks were identified before discharge from hospital. Four patients exhibited type II endoleaks. No local complications were observed.

### Mid-term follow-up

The mean follow-up period was 25.5 months (range 6.0–36). Follow-up data were available for 22, 19, 16, 12, and 7 patients at 6, 12, 24, and 36 months. No further interventions were required, and no subsequent cases of aortic rupture, endoprosthesis migration, or death were recorded.

## Discussion

High angulation and short length of the infrarenal aortic neck are predictors of failure of endograft sealing and fixation failure, which can result in type I endoleak, graft migration, and aneurysm rupture (18). As noted by Finotello et al. (14), the main reasons for the limited efficacy of standard endografts in these anatomical conditions are the surgeon’s inability to achieve precise, controlled deployment and the limited conformability of the endograft, which prevents orthogonal alignment with the main aortic axis.

The initial experience with the GECE shows promising results in patients with severe infrarenal neck angulation and short neck length. However, the available data remain limited and do not allow for a comprehensive assessment of the device’s performance in real-world clinical practice, as only two multicentre studies have been published to date (19, 20). Table 2 presents the published data on GECE use.

**Table 2:** The results of studies on the use of the Gore conformable endograft.

Author (year)	Single/multicentre	Patients	Angulation	Neck length (mm)	30 days Mort	30 -days Re-intraventions	Follow-up (months)
Bonvini et al. (2021) (13)	Single	5	89.4° (range 70°–122°)	18.4 (range 13–24)	No	No	5.2 months (range 1–11). No migration, no type IA endoleak and no graft kinking
Finotello et al. (2021) (14)	Single	12	Suprarenal (α) and infrarenal (β) neck angulation were, respectively, 28.9° (15.7°, 47.5°) and 75.0° (66.9°, 81.4°)	16.1 (10.7, 21.7)	No	No	At 1-month, 5 type II endoleaks with no need for reintervention. The overall mortality was 8.3%
Lee et al. (2022) (15)	Single	24	73.4° (range: 60°–90°)	<15 mm	no	no	12 months (*n* = 19)—no graft migration, no type I endoleak, type II endoleak were in 5 (26.3%) patients
Pitros et sl. (2023) (16)	Single	5	12°–90°	13–25 mm			
Mascoli. et al. (2023) (21)	Single	25	Median suprarenal aortic neck α angle of 38° (range: 5°–60°) and a median infrarenal aortic neck β angle of 70° (range: 60°–90°)	22 mm (range: 13–42)	No	No	3–30—no migration, no endoleak type I, no reintervention, no mortality unrelated to AAA
Rhee et al. (2023) (19)	Multicentre	80	35.7° ± 14.55° (range 3.0°–59.0°)	23.9 ± 13.07 (range 10.0–95.0)	No	No	No type I or type III endoleaks were detected on the 1-, 6-, or 12-month follow-up. No wire fractures, device migrations, or AAA ruptures were observed through the 12-month follow-up window
Elsharkawy et al. (2023) (20)	Multicentre	20	Infrarenal beta angulation 80 (70°–89°)	23 (18–28)	One case unrelated to the surgery	No	No endoleaks
Zuidema et al. (2023) (22)	Single centre	46	Infrarenal angulation 61.4° (44.6°–70.7°)	16.6 (12.5–26.4)	one	Two patients	0–30 months. One type III endoleak, two endograft migrations

### Technical success

As reported by the authors of the studies summarised in Table 2, the technical success rate of the procedure is in the range of 95%–100% (13–15, 21, 22). In the study by Elsharkawy et al. (20), technical success was achieved in 19 (95%) procedures, with 1 (5%) instance of type Ia endoleak. Several authors have noted that graft implantation required both intraoperative repositioning or correction of a type I endoleak using a cuff or ballooning of the proximal fixation zone. In our observations, the technical success rate of the procedure was 100%. Two rotational manoeuvres were performed during graft implantation in 12 patients, with reposition in three.

### Neck length and angulation

As shown in Table 1, the mean aneurysm neck length and angulation were generally within the instructions for use (13–16, 18–22). However, some patients still underwent EVAR despite having shorter necks and greater angulation than specified in the IFU. In our observations, EVAR was not performed in any case where the aneurysm had both a neck length <10 mm and an angulation >90%. In our opinion, deviating from the IFU on both parameters simultaneously is inadvisable. Nevertheless, a very short neck often requires chimney stenting of the renal artery and/or cuff implantation. Moreover, both short neck length and greater angulation are known risk factors for type I endoleak, reintervention, and late open conversion (23–25).

### Instructions of use/intraoperative complications

Some studies have shown that graft placement outside the IFU can still give good immediate results. For instance, Zuidema et al. (22) reported that 13 (28%) patients underwent graft implantation outside the IFU with good results. In our study, EVAR was performed outside the IFU in seven patients. In addition, due to very short necks, two patients required chimney stenting of the renal artery. During the postoperative period, no prosthesis migration or type I endoleak was observed. However, excessively short necks often require chimney stenting and/or cuff implantation. Bonvivni et al. (13) reported intraoperative type I endoleaks in two patients with the most severe infrarenal angulation. In both cases, final angiography confirmed patent renal arteries, but a type Ia endoleak was identified, attributed to inadequate graft apposition along the severely curved neck-aneurysm transition. These endoleaks were treated with angioplasty. Mascolli et al. (21) performed repositioning manoeuvres in 12 (80%) patients, and the active angulation system was used in 17 (68%) patients. The proximal sealing site was ballooned in all cases, with only one patient requiring an unplanned cuff. Similarly, Finotello et al. (14) reported complete aneurysm exclusion without any cases of type I endoleak or endograft kinking. Lee et al. (15) observed initial type Ia endoleaks in two patients and a type 1b endoleak in on patient, all managed successfully with balloon angioplasty. Type II endoleaks occurred in three patients but did not require immediate intervention. In their series, six patients required a single repositioning manoeuvre, one patient required two, and one patient required three repositions to optimise the landing position.

### Reinterventions

In our study, open conversion and graft explantation were required in one patient due to acute thrombosis on day 21. No reinterventions for endoleaks or graft migration were necessary in any patient. Similarly, Zuidema et al. (22) reported two cases requiring reintervention within 30 days, both due to thrombosis of the external iliac artery and common femoral artery on the ipsilateral side of the iliac branch.

### Mid-term/long-term follow-up

During the mean follow-up period in our study of 247.0 days (range 153.0–501.0), no type I or III endoleaks, graft migrations, or reinterventions were identified. Both the immediate and long-term postoperative outcomes, as supported by the studies presented in Table 2, showed good results. In the study by Zuidema et al. (22), one type III endoleak and two endograft migrations were reported during a follow-up period of 0–30 months (27). The newest 5-year studies have also demonstrated positive results; however, in that study, only 53 of the 80 patients were available for 5-year data analysis due to death and loss to follow-up. This indicates that true outcomes of those unavailable for follow-up may not have been captured and are therefore not represented in this dataset.

In addition, no local complications were observed after the use of the Manta closure device, consistent with findings from a multicentre study of patients who underwent large-bore percutaneous arterial access (26).

## Limitations of our study

Despite being a retrospective study on a heterogenous population, patient selection was not standardised, nor was the surgical technique.

## Conclusion

The use of the Gore excluder conformable endoprostheses is an effective method for treating abdominal aortic aneurysms with a hostile neck, showing good mid-term results. However, further research is needed to evaluate long-term outcomes. Since the design of the excluder has not significantly changed apart from its angulation capability, similar long-term durability to the standard excluder can be expected.

## Data Availability

The raw data supporting the conclusions of this article will be made available by the authors, without undue reservation.
